# Environment-friendly AgNWs/Ti_3_C_2_T_x_ transparent conductive film based on natural fish gelatin for degradable electronics

**DOI:** 10.3389/fchem.2022.973115

**Published:** 2022-08-05

**Authors:** Yuzhou Wang, Tao Wang, Yan Liu, Hong-Zhang Geng, Lianzhong Zhang

**Affiliations:** ^1^ Henan Engineering Technology Research Center of Ultrasonic Molecular Imaging and Nanotechnology, Henan Provincial People’s Hospital, People’s Hospital of Zhengzhou University, Zhengzhou, China; ^2^ College of Materials Engineering, Henan University of Engineering, Zhengzhou, China; ^3^ Tianjin Key Laboratory of Advanced Fibers and Energy Storage, School of Material Science and Engineering, Tiangong University, Tianjin, China; ^4^ Sinopec Petroleum Engineering Zhongyuan Corporation, Zhengzhou, China

**Keywords:** transparent conductive films, fish gelatin, degradable, silver nanowires, Ti_3_C_2_T_x_

## Abstract

Recently, the electronic waste (E-waste) has become the most serious environmental trouble because of the iteration of electronic products. Transparent conductive films (TCFs) are the key component of flexible electronic devices, so the development of devices based on degradable TCFs has become an important way to alleviate the problem of E-waste. Gelatin, one of the most prevalent natural biomacromolecules, has drawn increasing attention due to its good film-forming ability, superior biocompatibility, excellent degradability, and commercial availability at a relatively low cost, but has few applications in flexible electronics. Here, we report a method for preparing flexible TCF based on naturally degradable material-fish gelatin, in which silver nanowires and Ti_3_C_2_T_x_ flakes were used as conductive fillers. The obtained TCF has low roughness (RMS roughness = 5.62 nm), good photoelectric properties (Rs = 25.2 Ω/sq., T = ca.85% at 550 nm), strong interfacial adhesion and good degradability. Moreover, the film showed excellent application in the field of EMI shielding and green light OLED device. We believe that these TCFs will shine in the smart wearable field in the future.

## 1 Introduction

In the era of 5G internet, with the fast growth of flexible electronic technology, electronic gadgets prepared based on transparent conductive films (TCFs) along with organic light-emitting diodes (OLED) (Koo et al., 2010), antennae (Bengio et al., 2019), solar cells (Li et al., 2010), etc., have significantly enhanced and expanded people’s daily life. However, the service life of electronic devices is limited, it will invariably be damaged and lose functionalities during usage, resulting in electronic waste (E-waste) (Islam et al., 2020). The global annual output of E-waste is about 40 metric tons, accounting for 5% of the total global solid wastes (Hazra et al., 2019). E-wastes are difficult to biodegrade and contain large amounts of harmful metals (Cd, Hg, Pb, Cr) and hazardous chemicals, which can penetrate plants and animals through soil and groundwater and have long-term consequences on the entire ecological system. E-waste has risen to the top environmental issue in recent years.

To alleviate the pressure of E-waste on environmental protection, many scholars have proposed to use naturally degradable materials to replace traditional petroleum-derived plastics like poly dimethyl siloxane (PDMS) (Wang D. et al., 2020), polyethylene terephthalate (PET) (Wang T. et al., 2020), polyethylene (Zardetto et al., 2011), polyimide (Bae et al., 2016), polyurethane (Wang, et al., 2019), etc., to prepare “green” flexible wearable electronic devices. Tao et al. (2017) made cellulose nano paper from sugarcane bagasse for field-effect transistors; Yang et al. (2020) novelty use a self-supporting natural dextran membrane as the gate dielectric to achieve an ultra-flexible and degradable organic synaptic transistor; Xia et al. (2020) presented a technique for producing optically transparent wood by changing the wood slignin structure with a solar-assisted chemical brushing technique, and the transparent wood film may be employed as an electronic device substrate. Although the transparent substrates obtained by the aforesaid methods can be utilized to prepare green electronic devices, the costs were high, the processes were complicated, and the transparency of the prepared films was poor. Therefore, a simple method to prepare low-cost and high-performance transparent substrates has emerged as a critical link to solve the problem.

Gelatin (Etxabide et al., 2016), one of the most prevalent natural biomacromolecules, has been widely used in recent years because of its attributes such as good film-forming ability, superior biocompatibility, excellent degradability, and commercial availability at a relatively low cost. The traditional raw materials of gelatin production are the skins and bones of pigs and cattle. However, due to the religious culture and the risk of disease transmission, fish gelatin (FG) has gradually become an alternative to mammalian gelatin (Hosseini et al., 2013). FG could be obtained from fish scales, which are inedible wastes that account for about 3% of the annual production of the 70.5 million metric tons of fish, making it highly cost-efficient and sustainable; these advantages make FG a perfect candidate for transparent substrates (Zhang J. et al., 2020). However, FG-based films have relatively weak mechanical properties which can be improved by blending with other biodegradable polymers which have gradually become an innovative approach to improve the cost-performance ratio of the resulting films. Poly (vinyl alcohol) (PVA) has favorable biodegradability, good mechanical properties, and excellent film-forming ability, which can be mixed with FG to improve the physical properties of the substrates due to the formation of hydrogen bond between themselves (Ghaderia et al., 2019).

Currently, indium tin oxide (ITO) (Kang et al., 2020) has become a popular conductive material for electronic applications, whereas it has a lot of limitations, including the scarcity of indium, the high-temperature deposition process, and the fragile nature, all of which limit its widespread application in next-generation flexible electronic devices. A significant amount of work has been expended in the search for optimal replacement conductive materials, such as carbon nanotubes (CNTs), graphene, metal nanowires, conductive polymers, etc. (Jing et al., 2020; Zhao et al., 2020; Zhu et al., 2020). Among these candidates, silver nanowires (AgNWs) have piqued the interest of researchers because to their outstanding intrinsic electrical conductivity, superb optical transparency, high flexibility, and low processing temperature. However, owing of the substantial contact resistance between nanowires, pure AgNWs TCFs often display low conductivity, inadequate electrical stability, and poor mechanical flexibility, severely limiting their applicability (Chen et al., 2020).

Since the first discovery of the two-dimensional (2D) nanomaterial Ti_3_C_2_T_x_ in 2011 (Liu et al., 2019), MXenes (transition metal carbides, carbonitrides, and nitrides) have been widely used in energy storage devices, water desalination, and electromagnetic radiation protection due to its superior electrical conductivity, hydrophilic surfaces, and large specific surface area. Nevertheless, the sheet-like structure of MXenes makes it easy to restack and aggregate, which may reduce accessible surfaces and slow transports of electrons and ions (Liu et al., 2018).

For the preparation of high-performance TCFs and applications in flexible wearable optoelectronic devices, it is necessary to combine the 1D AgNWs and 2D MXene nanosheets to build a new high-efficiency 3D conductive network. To overcome the drawbacks that the TCFs prepared by the traditional solution coating method are easy to peel off from the substrate, we integrated the 3D conductive network onto the FG/PVA substrate by a simple solution method to obtain a new type of “green” TCFs. Interestingly, the TCFs still exhibited good conductivity and transparency, and the interfacial adhesion of the film could be improved, which were of great significance for the long-term and stable use of electronic devices based on the films.

Here, we successfully fabricated the new type “green” degradable AgNWs/Ti_3_C_2_T_x_-FG/PVA TCFs by a simple method. The films consist of a high conductivity conductive network composed of AgNWs and Ti_3_C_2_T_x_ flakes. The degradable FG/PVA film with low cost, high light transmittance, and good mechanical strength replaces the traditional petroleum derivative materials as the substrate. The excellent performance of the film shows its potential application value as EMI shielding film and optoelectronic device. Besides, the strategy to develop “green” electronics based on the TCFs may pave a way for the coming of sustainable development and intelligent era in the future.

## 2 Experimental

### 2.1 Materials

Ti_3_AlC_2_ powder (MAX, 400 mesh) was purchased from Jilin Province 11 Technology Co., Ltd. Concentrated hydrochloric acid (HCl, 36.5%), lithium fluoride (LiF, AR, 99%), sodium hydroxide (NaOH, AR, 95%), silver nitrate(AgNO_3_, AR, 99%),poly(vinyl pyrrolidone) (PVP; MW = 13,00,000), polyvinyl alcohol (PVA) (Mw of 1,700, 87%–89% hydrolyzed), and aluminum (Al, AR, 99%) were purchased from Shanghai Mackin Biochemical Co., Ltd. N,N′-bis (naphthalene-1-yl)-N,N′-bis (phenyl) benzidine (NPB) and Hydroxyquinoline aluminum (Alq3) (PLT401011G) were purchased from Xi’an Baolaite Optoelectronics Technology Co., Ltd. Fish scales were collected from the supermarket. The polyethylene terephthalate (PET; thickness = 0.15 mm) substrate was procured from Carbon Star Technology (Tianjin) Co., Ltd. Deionized (DI) water was used for all experiments.

### 2.2 Preparation of high-quality few-layer Ti_3_C_2_T_x_ by the minimally intensive layer delamination method

The Ti_3_C_2_T_x_ flakes were obtained by the minimally intensive layer delamination (MILD) method (Alhabeb et al., 2017). First, 40 ml of 9 M HCl solution and 3.2 g of LiF powders were added into a tetrafluoroethylene beaker and stirred evenly, then 2 g of Ti_3_AlC_2_ powders were gradually added to the etchant solution under slow stirring at 40°C for 24 h. After the reaction, DI water was added to the beaker, and the resulting solution was repeatedly washed at 3,500 rpm for 5 min until a stable dark green supernatant with a pH of 6 was obtained. Subsequently, the obtained multi-layer Ti_3_C_2_T_x_ solution was shaken by hand (200 times) and centrifuged at 3,500 rpm for 5 min. In this process, the solution after each centrifugation did not need to be poured out and continued to be manually shaken. After about 10 cycles, the exfoliation process ended; the above solution was then centrifuged at 3,500 rpm for 30 min to collect the solution. The obtained solution after centrifugation was a few-layer of Ti_3_C_2_T_x_, and the precipitate was unreacted Ti_3_AlC_2_ and Ti_3_C_2_T_x_ with multilayer or incomplete peeling.

To remove the excess LiF impurities in the solution, an appropriate amount of 1 M HCl solution was added into the collected solution, stirred at room temperature for 10 min, and then centrifuged at 3,500 rpm for 5 min until the pH of the solution was 6 to obtain high-quality Ti_3_C_2_T_x_ solution (The concentration of Ti_3_C_2_T_x_ solution prepared in this article was ∼2 mg/ml).

### 2.3 Synthesis of AgNWs

The AgNWs were prepared by the polyol method as reported in our previous work (Zhu et al., 2020). The concentration of AgNWs solution prepared in this article was ∼3 mg/ml. The fabricated AgNWs and AgNWs film were shown in Supplementary Figure S1.

### 2.4 Fabrication of FG/PVA films

The FG solution was fabricated by a simple method. Firstly, the fish scales collected from the market were washed with water to remove the impurities and dried in the sun and then washed with 0.1 M NaOH and 1 M HCl to remove the minerals. After that, the fish scales and water were added to a sealed beaker at a mass ratio of 1:6 kept at 80°C for 6 h. Then the FG solution was filtered through gauze and centrifuged at 5,000 rpm for 20 min to remove the residue in the solution. Finally, the FG solution with a solids content of about 5 wt.% was obtained. Besides, PVA solution (5 wt.%) was prepared by dissolving PVA in distilled water at 90°C under vigorous stirring for 4 h. The different ratio FG and PVA solutions (1:0, 1:0.5, 1:1, 1.5, 1:2) were mixed and stirred at 80° for 30 min, and glycerin was added to the mixture as a plasticizer. Finally, the FG/PVA solutions were poured and dried in the mold at room temperature for 24 h to obtain the FG/PVA films.

### 2.5 Preparation of the AgNWs/Ti_3_C_2_T_x_-FG/PVA films

**FIGURE 1:**
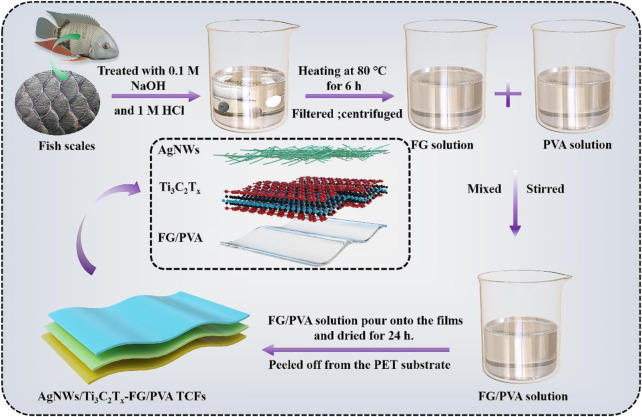
The preparation process of the AgNWs/Ti_3_C_2_T_x_-FG/PVA film.

The preparation process of the AgNWs/Ti_3_C_2_T_x_-FG/PVA film was shown in Figure 1. The PET substrates were cut into 4 × 4 cm^2^ size, to remove the contaminants on PET surfaces, it was immersed in acetone and ultrasonic for 30 min, then dried and set aside. Besides, the pristine Ti_3_C_2_T_x_ solution and AgNWs solution were diluted to 1 and 1.5 mg/ml, respectively to ensure the smooth spraying process.

The AgNWs/Ti_3_C_2_T_x_-PET films were prepared as followed: At first, PET substrates were fixed on the heating plate (the heating temperature was set to 60°C). Next, the Ti_3_C_2_T_x_ solution and AgNWs solution with the volume ratio of 0.5:1 were gradually sprayed onto the PET substrates through the airbrush (Fuso Seiki Co. Ltd., Japan) under certain spray conditions (the outlet pressure of the airbrush was 0.5 MPa, and the distance between the airbrush nozzle and the heating table was 25 cm) to avoid the coffee-ring effect. A series of AgNWs/Ti_3_C_2_T_x_-PET TCFs with different sheet resistance were fabricated by varying the spray-coating volume. In the end, the AgNWs/Ti_3_C_2_T_x_-PET films were dried in a vacuum oven for later processing.

For AgNWs/Ti_3_C_2_T_x_-FG/PVA films, the AgNWs/Ti_3_C_2_T_x_-PET films were fixed in the stainless-steel molds; and then the same amount of FG/PVA solution with different ratios were poured onto the films and dried at room temperature for 24 h. After drying, the AgNWs/Ti_3_C_2_T_x_-FG/PVA films were peeled off from the PET substrate and stored in a vacuum glove box for later use.

### 2.6 Measurement of EMI shielding performance

The EMI shielding test was using an AgilentN5244A vector network analyzer in 8–12 GHz (X-Band) with sample sizes of 22.58 × 10.14 mm^2^ based on the waveguide method.

### 2.7 Fabrication of OLEDs using AgNWs/Ti_3_C_2_T_x_-FG/PVA TCFs as anode

The OLEDs which using AgNWs/Ti_3_C_2_T_x_-FG/PVA TCFs as anode was prepared as reported in our previous work (Wang D. et al., 2020). The OLED structures in this study were AgNWs/Ti_3_C_2_T_x_-FG/PVA/PEDOT: PSS (20 nm)/NPB (40 nm)/Alq3 (60 nm)/LiF (1 nm)/Al (80 nm). All of the organic materials were thermally evaporated onto the patterned anodes using a thermal evaporation coating system at a pressure of approximately 7 × 10^−4^ Pa.

### 2.8 Characterization

The obtained samples were characterized by the transmission electron microscopy (TEM, Hitachi H-800), X-ray diffraction (XRD, D8 Discover, Bruker, Germany), X-ray photoelectron spectroscopy (XPS, Thermo Fisher, United States), UV-Vis spectrophotometer, Field-emission scanning electron microscopy (FE-SEM) (Hitachi S-4800), Fourier-transform infrared spectroscopy (FT-IR,TENSOR 37, Germany), Testing machine (TVT-300Xp, TexVol Instruments, Viken, Sweden), the sheet resistance device (Keithley 2,700 multi-meter), ultraviolet photoelectron spectroscopy (UPS, Thetaprobe Thermo). Adhesive tape (Scotch Magic Tape, 3 M, United States) was utilized to evaluate the adhesion of the films. The performance of the OLEDs was characterized by an optical detection system for OLED display panels developed by Suzhou Fstar Scientific Instrument Co., Ltd. Electromagnetic parameters of composite films were obtained using a network analyzer (Agilent Technologies N5244A) in the frequency ranges of 8.2–12.4 GHz (X-band) at room temperature.

## 3 Results and discussion

**FIGURE 2:**
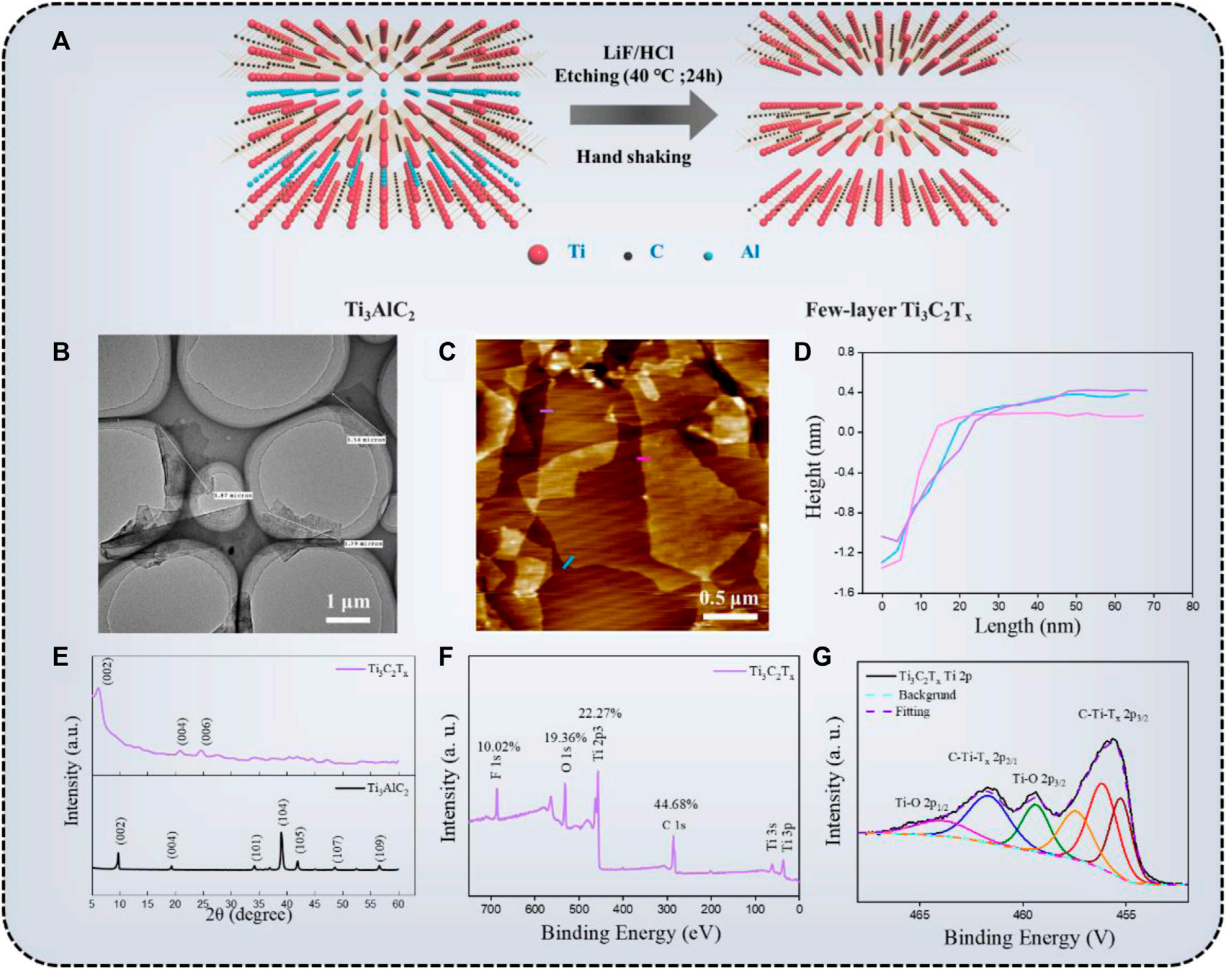
**(A)** The schematic illustration of the preparation of the Ti_3_C_2_T_x_ *via* the MILD method; TEM micrograph **(B)**, AFM image **(C)**, thickness **(D)**, XRD spectra **(E)**, and XPS spectra **(F,G)** of the Ti_3_C_2_T_x_ flakes.

The schematic illustration of the preparation of the Ti_3_C_2_T_x_ *via* the MILD method and property characterizations are shown in Figure 2. In Figure 2A, the Ti_3_C_2_T_x_ flakes were obtained by the MILD method. The MAX phase-Ti_3_AlC_2_ powder was etched in the HCl/LiF solutions at 40°C for 24 h. The Al layers were successfully etched by the *in-situ* formed HF. Besides, themulti-layered-Ti_3_C_2_T_x_ sheets were further exfoliated to a few-layer-Ti_3_C_2_T_x_ by manual hand-shaking (no sonication). From the TEM image (Figure 2B) we can see, the average lateral size of Ti_3_C_2_T_x_ nanosheets was 1.7 ± 0.2 μm, and these flakes have clear and neat edges without visual holes, indicating that the MILD method can overcome the shortcomings of excessive flake defects caused by the sonication (Weng et al., 2018). AFM test (Figures 2C,D) was another method to characterize the quality of Ti_3_C_2_T_x_. According to Ahn’s report (Ahn et al., 2020), the thickness of the single Ti_3_C_2_T_x_ flake was about 1 nm, but the average thickness of Ti_3_C_2_T_x_ sheets we obtained was ∼1.5 nm. This was attributed to our AFM measurement’s high sensitivity, which identifies surface functional groups and/or adsorbed molecules on and under the flake. The crystal structure of Ti_3_AlC_2_ and Ti_3_C_2_T_x_ particles were further confirmed by XRD patterns (Figure 2E). Compared with Ti_3_AlC_2_, many diffraction peaks of Ti_3_C_2_T_x_ disappeared after the Al layers were etched. Moreover, the (002) peak shifted from 9.7° to 6.2°, and the corresponding c-lattice spacing of the Ti3C2Tx was 25.1 Å, which is in agreement with literature data (Zhou et al., 2019). The chemical composition of the Ti_3_C_2_T_x_nanosheets was examined by XPS spectra. The Ti, C, F, and O were discovered in the survey spectra, while the F and O elements were acquired from the etching agent. The XPS spectrum of Ti_2P_ was shown in Figure 2G, the peaks at 455.7 eV (sp_3/2_) and 461.6 eV (sp_1/2_) represent a contribution from Ti-C bonds, and the peaks at 459.3 and 463.6 eV correspond to Ti-O bonds (Ahn et al., 2020). The weak intensity of the Ti-O bonds means the prepared Ti_3_C_2_T_x_ flakes have no sign of oxidation. According to the above evidence, it showed that the few-layer, uniform diameter distribution, and unoxidized Ti_3_C_2_T_x_ flakes were successfully prepared.

**FIGURE 3:**
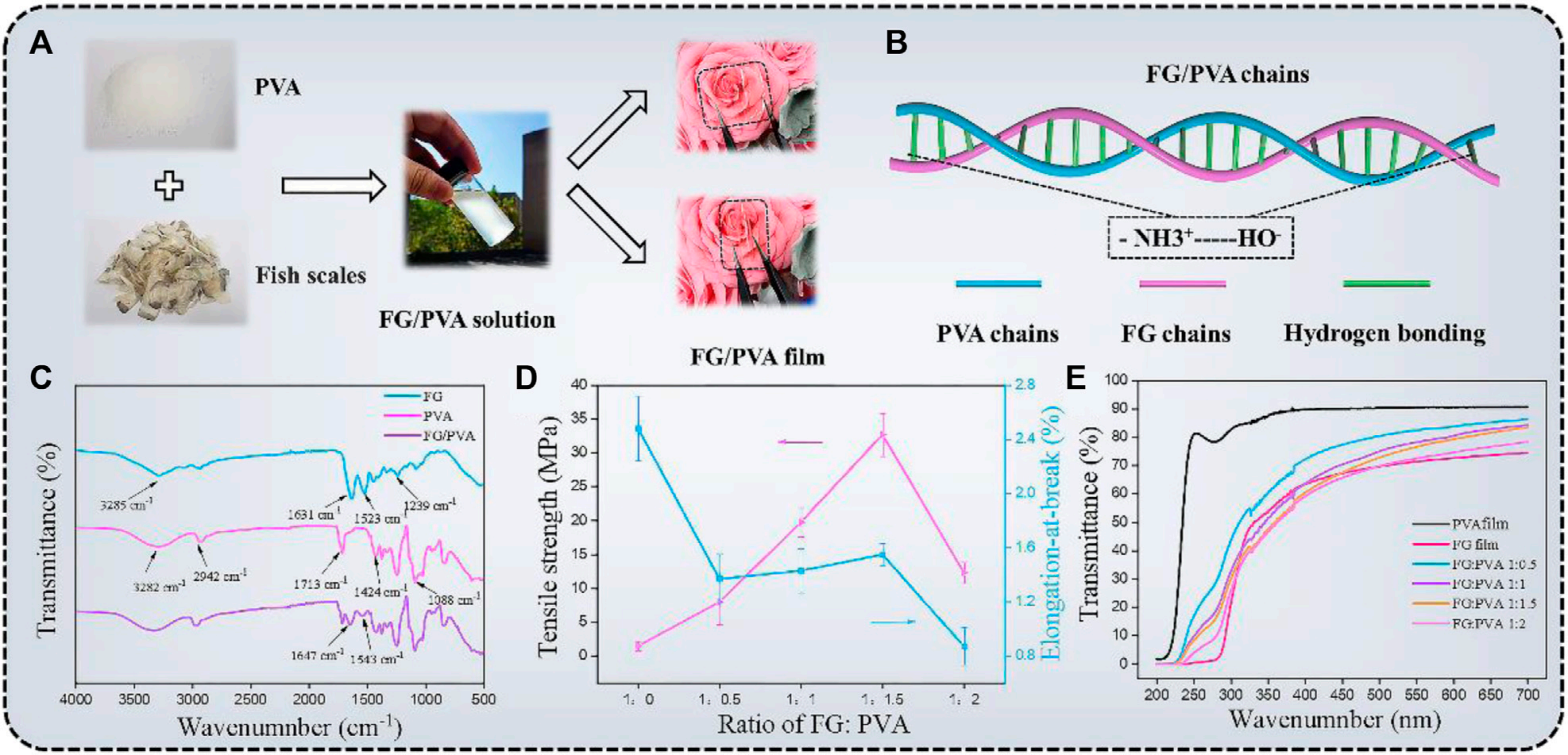
**(A)** Preparation process of the FG/PVA film. **(B)** The interaction mechanism between FG and PVA. **(C)** FT-IR spectra of FG, PVA, and FG/PVA films. The values of the tensile strength (TS), elongation-at-break (EB), and transmittance of different films **(D,E)**.

The FG/PVA films were synthesized by the solution casting method. As shown in Figure 3A, FG was extracted from natural fish scales and then mixed with PVA (glycerin was added as a plasticizer to improve the processing property of the film), then the mixture solution was poured into the mold and dried at room temperature to obtain FG/PVA film. Figure 3B was the schematic of the interaction mechanism between the FG and PVA chains. With the addition of PVA to FG, the two chains could entangle with each other and form hydrogen bonds, resulting in a hybrid film with better performance. The interactions between PVA and FG were analyzedby FT-IR measurement (Figure 3C). The four typical peaks in the FT-IR spectra of FG film were located around 3,285, 1,631, 1,523, and 1,239 cm^−1^, which were related to amide A (N-H stretching), amide-I (C=O stretching and the stretching vibration of O-H groups), amide-II (N-H bending), and amide-III (C-N stretching and N-H bending or vibrations of CH_2_ groups in glycine), respectively (Etxabide et al., 2016). In the spectrum of PVA film, the strong and broad absorption peak at about 3,282 cm^−1^ was attributed to the stretching vibration of hydroxyl groups (O-H). Besides, the absorption peaks at 2,942, 1,713, 1,424, and 1,088 cm^−1^ were corresponding to C-H stretching, C=O stretching, O-H bending, and C-O stretching, respectively (Zhang X. et al., 2020). As for the FT-IR spectrum of FG/PVA film,the characteristic peaks of FG and PVA appeared simultaneously in the spectrum of the hybrid film, which indicated that the FG was compatible with PVA. In addition, the position of the amide-II peak in the FG/PVA film shifted from 1,523 cm^−1^ to 1,543 cm^−1^ and the amide-I peak shifted from 1,631 cm^−1^ to 1,647 cm^−1^. The formation of various types of intra/intermolecular hydrogen bonds between N-H and O-H groups was considered to be the mechanism for this (Ghaderia et al., 2019).

The mechanical strength and flexibility were important index to evaluate the film performance. The values of the tensile strength (TS), elongation-at-break (EB) of different films were exhibited in Figure 3D. FG film (FG:PVA = 1:0) has low TS (1.5 MPa ± 0.72 MPa) but high EB (248% ± 24%). To enhance the mechanical strength of the film, PVA was introduced into FG to fabricate the FG/PVA film. The development of intermolecular hydrogen bonding between FG and PVA increased the TS of the hybrid film to 32.67 MPa ± 3.16 MPa (FG:PVA = 1:1.5) with the addition of PVA. Excess PVA molecules were present in free form in the hybrid film as the fraction of PVA continued to rise, resulting in a reduction in tensile strength (Uranga et al., 2016).

A highly transparent substrate was also a crucial prerequisite for the preparation of high-performance optoelectronic devices. The transmittance of the hybrid film with different PVA addition amounts was investigated by UV-vis spectrometer (Figure 3E). The optical transmittance at 550 nm of different films (FG; FG/PVA 1:0.5; FG/PVA 1:1; FG/PVA 1:1.5; FG/PVA 1:2; PVA) were found to be 82.47%, 78.19%, 76.52%, and 72.47%, respectively. Considering the mechanical properties and transmittance of the film, the optimal ratio of FG: PVA was 1:1.5. The FG: PVA film, in particular, remained undamaged after being submerged in several organic solvents for 24 h, demonstrating high chemical stability (Supplementary Figure S2) (Zhang X. et al., 2020).

**FIGURE 4:**
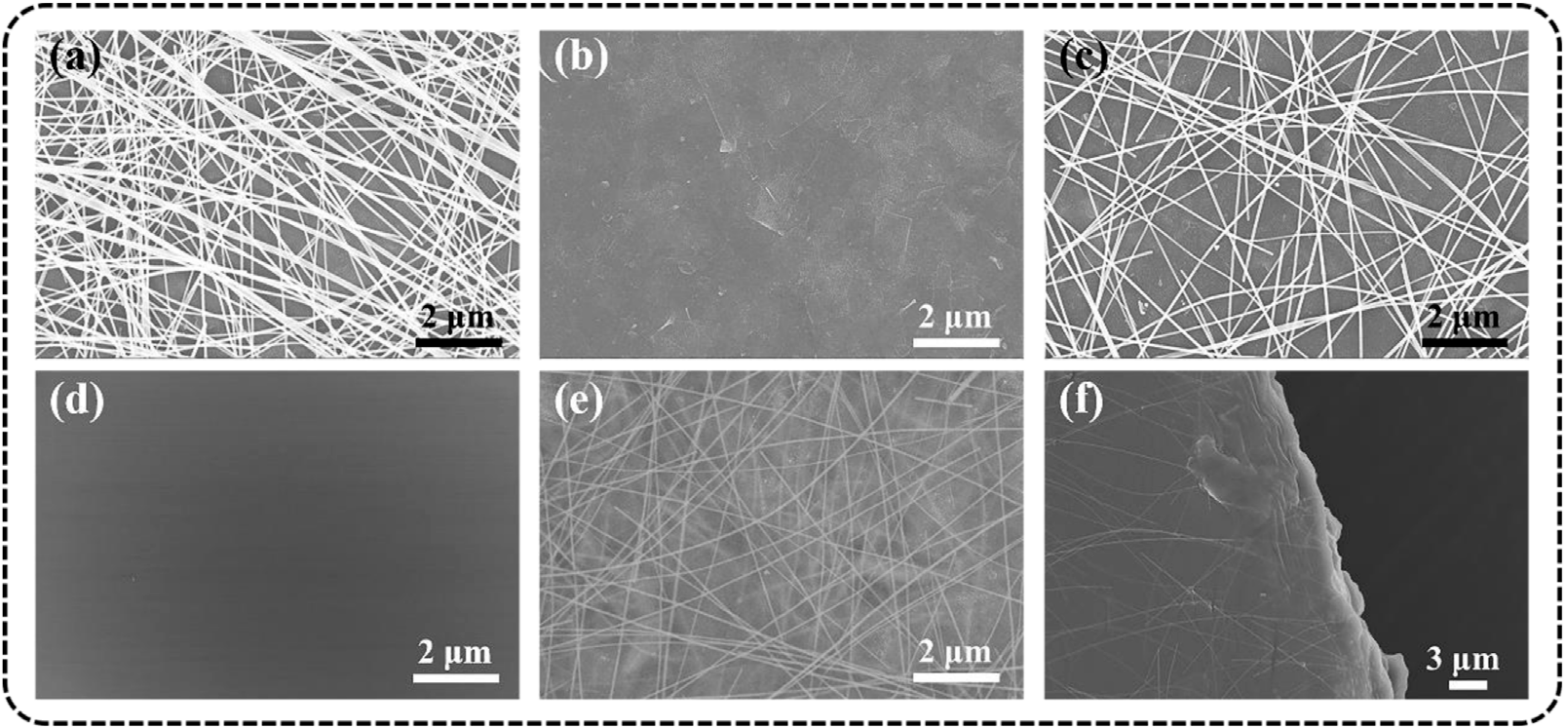
SEM images of different films: **(A)** AgNWs-PET, **(B)** Ti_3_C_2_T_x_ -PET, **(C)** AgNWs/Ti_3_C_2_T_x_-PET, **(D)** FG/PVA, **(E)** surface, and **(F)** profile of AgNWs/Ti_3_C_2_T_x_-FG/PVA.

The surface morphology of different TCFs was characterized by SEM. All the TCFs (AgNWs-PET, Ti_3_C_2_T_x_ -PET, and AgNWs/Ti_3_C_2_T_x_ -PET) were prepared by the spray-coating method, which makes the conductive inks evenly coated on the substrate; moreover, they all exhibited clean surfaces without impurities. As for AgNWs-PET film (Figure 4A), the AgNWs stacked loosely and connected simply, and the junctions exhibited distinct outlines (Liang et al., 2017). When the AgNWs were deposited on the Ti_3_C_2_T_x_ layer (Figure 4B), the “voids” in the AgNWs conductive network would be filled (Figure 4C) and the elemental maps of Ag, Ti, and C on the surface prove the uniform distribution of Ti_3_C_2_T_x_ and AgNWs (Supplementary Figure S3). In addition, 1D AgNWs and 2D Ti_3_C_2_T_x_ flakes could form a dense 3D conductive network, which not only provides more carrier transmission paths to reduce the resistance of the film but also reduces the roughness of the film. From the SEM image of FG/PVA film, we can know that the hybrid film had smooth and flat surfaces, was free of pores, and had good structural integrity (Figure 4D), which proved the good compatibility between FG and PVA and consistent with the previous report (Ghaderia et al., 2019). Through a simple solution casting method, we successfully integrated the 3D conductive network onto the surface of the substrate and obtained the novel TCFs. In the SEM images of AgNWs/Ti_3_C_2_T_x_-FG/PVA film (Figures 4E,F), the AgNWs/Ti_3_C_2_T_x_ layer was partially encased in the cured FG/PVA film and the conductive layer kept intact. The changes of the roughness, conductivity and mechanical properties of the AgNWs/Ti_3_C_2_T_x_-FG/PVA film compared with the AgNWs/Ti_3_C_2_T_x_ -PET film will be discussed as followed.

**FIGURE 5:**
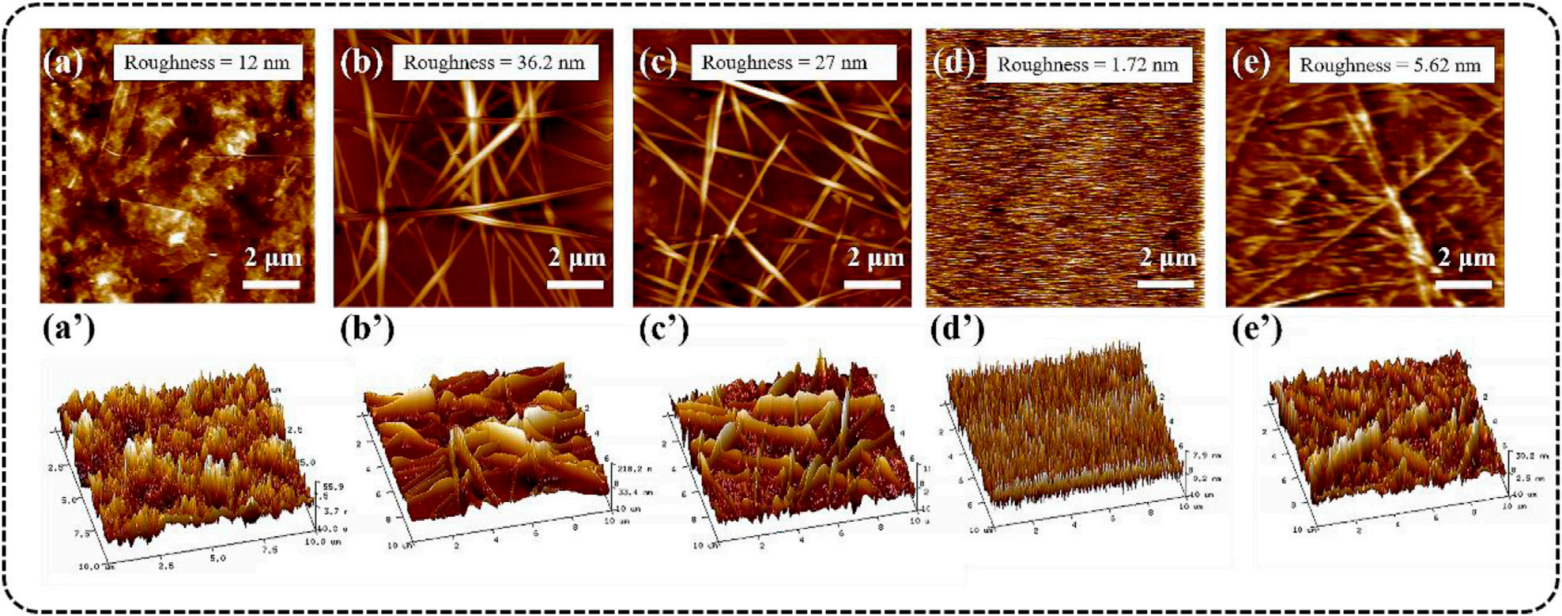
AFM (up) and 3D AFM (down) images of different TCFs: **(A-A’)** Ti_3_C_2_T_x_-PET, **(B-B’)** AgNWs-PET, **(C-C’)** AgNWs/Ti_3_C_2_T_x_-PET, **(D-D’)** FG/PVA, (**E-E’)** AgNWs/Ti_3_C_2_T_x_-FG/PVA.

The tapping mode AFM (2D) images of different TCFs were shown in Figures 5A–E, and the 3D AFM images (Figures 5A–E) corresponding to the height patterns further visually confirmed the surface features of the films. The root means square (RMS) roughness of the Ti_3_C_2_T_x_-PET and AgNWs-PET film was 12.0 and 36.2 nm, respectively. From their images, it can be seen that the Ti_3_C_2_T_x_flakes and AgNWs were evenly distributed on the PET substrate and overlap each other, which was consistent with the SEM results. As the Ti_3_C_2_T_x_ nanosheet may cover the “holes” in the AgNWs networks, the roughness of the film (AgNWs/Ti_3_C_2_T_x_-PET) reduced to 27.0 nm. For optoelectronic devices using TCFs as an anode, the main problem was the TCFs′ high surface roughness, which might allow these materials to penetrate through the active layer to the counter electrode, resulting in short circuits in the devices. To solve this problem, we transferred the conductive layer onto the flat FG/PVA film (RMS roughness = 1.72 nm) surface. This method can effectively minimize the roughness of the film while maintaining the integrity of the coating. The RMS roughness of the AgNWs/Ti_3_C_2_T_x_-FG/PVA film was only 5.62 nm, which has a broad application prospect in the field of flexible wearable devices.

**FIGURE 6:**
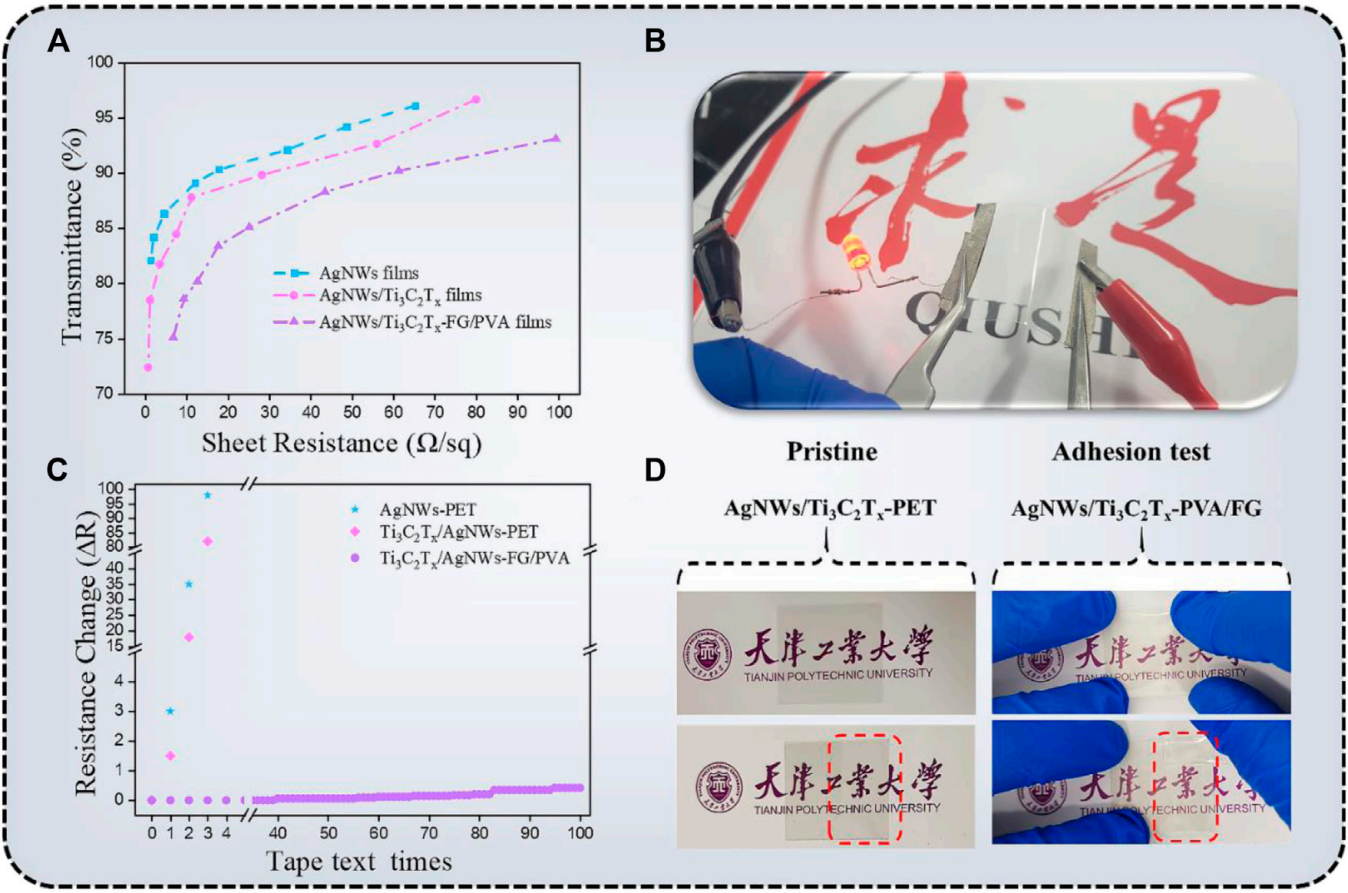
**(A)** The relationship between the transmittance (at 550 nm) and sheet resistance of AgNWs-PET, AgNWs/Ti_3_C_2_T_x_-PET, AgNWs/Ti_3_C_2_T_x_-FG/PVA TCFs; **(B)** Photograph of the AgNWs/Ti_3_C_2_T_x_-FG/PVA TCF connects to a LED; **(C)** The sheet resistance change of AgNWs-PET, AgNWs/Ti_3_C_2_T_x_-PET, AgNWs/Ti_3_C_2_T_x_-FG/PVA films as a function of tape test times; **(D)** Photograph of the AgNWs/Ti_3_C_2_T_x_-PET and AgNWs/Ti_3_C_2_T_x_-FG/PVA films after tape test.

Among several performance indicaters of TCFs, photoelectric properties are the most critical physical factors in photovoltaic devices.The transmittance (T) at 550 nm (PET substrate was used as a blank contrast sample) as a function of sheet resistance (Rs) of different TCFs was plotted in Figure 6A. All the films were obtained by evenly spraying the conductive inks onto the substrates, which can ensure the formation of the conductive networks, and free from notorious percolation problems. The AgNWs-PET film shows excellent photoelectric properties (T < 90%; Rs < 20 Ω/sq) because AgNWs have high electronic density, excellent conductivity, and high transmittance in visible light. According to Dillon’s report (Dillon et al., 2016), MXenes have metallic conductivity (the electrical conductivity of Ti_3_C_2_T_x_ sheets was calculated to be 4,762.5 S/cm in this paper), which is a result of metal-like high free-electron density and a sheet-like structure of individual nanosheets. The characteristic resistance of the Ti_3_C_2_T_x_-PET film was 432.6 Ω/sq. with an opticaltransmittance of 75.2% at 550 nm as shown in Supplementary Figure S4.

According to the SEM and AFM analysis, it can be seen that for this 3D conductive network (AgNWs/Ti_3_C_2_T_x_), on the one hand, the Ti_3_C_2_T_x_flakes filling the holes in AgNWs′ network can minimize the roughness of the film; on the other hand, this nanosheet with remarkable conductivitycan create several carriers transport channels, lowering the film’s surface resistance(AgNWs/Ti_3_C_2_T_x_-PET; Rs = 8.5 Ω/sq., T = ca.85% at 550 nm). Compared with AgNWs/Ti_3_C_2_T_x_-PET film, the resistance of AgNWs/Ti_3_C_2_T_x_-FG/PVA film has a certain increase, and its characteristic resistance was Rs = 25.2 Ω/sq., T = ca.85.1% at 550 nm. The reason for the increase of resistance can be attributed to the fact that part of the AgNWs/Ti_3_C_2_T_x_ conductive layer embedded in the FG/PVA film, which may lead to the interruption of some conductive paths. Figure 6B shows that the AgNWs/Ti_3_C_2_T_x_-FG/PVA film was put into a circuit connected with a LED light, showing its good photoelectric performance and flexibility.

Low sheet resistance and good transmittance were both necessary for viable optoelectronics applications. The performance of AgNWs/Ti_3_C_2_T_x_-FG/PVA film was evaluated using the figure of merit (FoM), which is defined as the ratio of electrical conductivity (*σ*_*dc*_) to optical conductivity (*σ*_*op*_). The FoM was estimated using the following formula (Zhao et al., 2020):

$$\frac{\sigma_{dc}}{\sigma_{op}}=\left[\frac{188.5\left(\Omega \right)}{R_{s}}\frac{\sqrt{T}}{\left(1-\sqrt{T}\right)}\right]$$ (1)

where *T* is the TCFs′ transmittance (at 550 nm), and *Rs* is their sheet resistance. Table 1 showed the values of *σ*_*dc*_ */σ*_*op*_ for the AgNWs/Ti_3_C_2_T_x_-FG/PVA films; all the samples show excellent performance.

**TABLE 1:** Optoelectrical properties of the AgNWs/Ti_3_C_2_T_x_-FG/PVA films.

Samples	1	2	3	4	5	6	7	8
*T*(%)	93.1	90.2	88.3	85.1	83.4	80.2	78.6	75.1
*R*_*s*_ (Ω/sq.)	99.3	61.3	43.5	25.2	17.7	12.7	9.3	6.8
*σ*_*dc*_ */σ*_*op*_	52.2	58.1	67.5	89.1	112.1	127.3	158.4	180.1

For the various devices based on TCFs, good adhesion is essential to ensure a long-term and efficient use of devices. Unfortunately, the coatings frequently fall off due to insufficient interfacial adhesion between the conductive layers and substrates, along with cohesion within the TCFs materials, which is the most urgent problem to be solved. Hence, we tested the interface adhesion of the AgNWs-PET, AgNWs/Ti_3_C_2_T_x_-PET, and AgNWs/Ti_3_C_2_T_x_-FG/PVA films with the 3M tape to characterize the adhesion properties. Figure 6C showed the sheet resistance change after tape adhesion trials which was calculated using the equation (Da et al., 2017):

$$\Delta R=\left(R-R_{0}\right)∕R_{0}$$ (2)

where *R*_*0*_ is the original sheet resistance value; *R* is the sheet resistance after the test. It is clear to see that the sheet resistance of AgNWs-PET and AgNWs/Ti_3_C_2_T_x_-PET film was greatly changed. Although the AgNWs/Ti_3_C_2_T_x_ network has great conductivity and mechanical flexibility, it also has drawbacks including excessive surface roughness and poor substrate adhesion. Compared with AgNWs-PET and AgNWs/Ti_3_C_2_T_x_-PET, the conductive layer of AgNWs/Ti_3_C_2_T_x_-FG/PVA was not simply deposited on the substrate, but partly imbedded into the FG/PVA film. This imbedded structure makes the TCFs have strong adhesion to ensure the integrity of the coating during the testing process, and the film transmittance almost remains unchanged.

The images of AgNWs/Ti_3_C_2_T_x_-PET and AgNWs/Ti_3_C_2_T_x_-FG/PVA films before and after the adhesion test intuitively show the difference of adhesion properties between these two type of film as shown in Figure 6D. It is obvious that the coating of theAgNWs/Ti_3_C_2_T_x_-PET film was readily detached from PET substrates after the test, while the AgNWs/Ti_3_C_2_T_x_-FG/PVA film without cracking or delamination. Besides, the mechanical stability of the AgNWs/Ti_3_C_2_T_x_-FG/PVA was estimated by bending test, and theresistance of the film remained stable after 1,000 bending times, indicating good flexibility of the film (Supplementary Figure S5).

**FIGURE 7:**
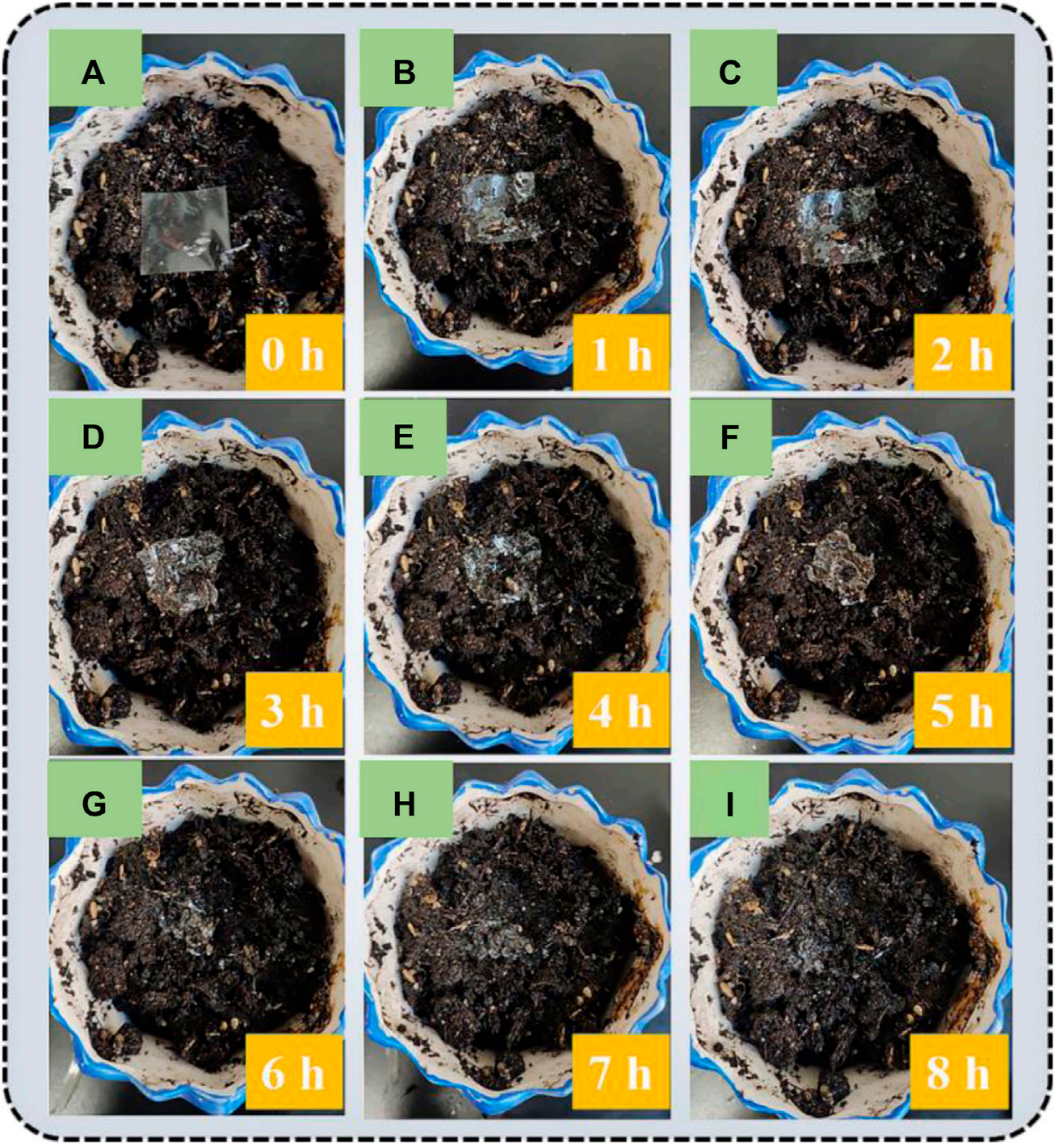
The degradable test of AgNWs/Ti_3_C_2_T_x_-FG/PVA film. **(A-I)** are corresponding to the picture of the film after 0-8h of degradation.

As can be seen from the above discription, AgNWs/Ti_3_C_2_T_x_-FG/PVA film has a fairly flat surface, good photoelectric performance, excellent adhesion, and flexibility. However, substituting degradable materials for typical petroleum derivative substrates has been considered as a vital component of degradable “green” devices. Both FG and PVA have admirable biodegradability, which gives degradability to AgNWs/Ti_3_C_2_T_x_-FG/PVA film and devices based on the film; and it will largely alleviate environmental pollution caused by electronic equipment. The degradation test was carried out by burying the film in the soil and observing it every one hour (Verma et al., 2018).We found that the film gradually degraded as shown from Figures 7A–H, and completely degraded after 8 h (Figure 7I), which can achieve “no harm” to the environment.

**FIGURE 8:**
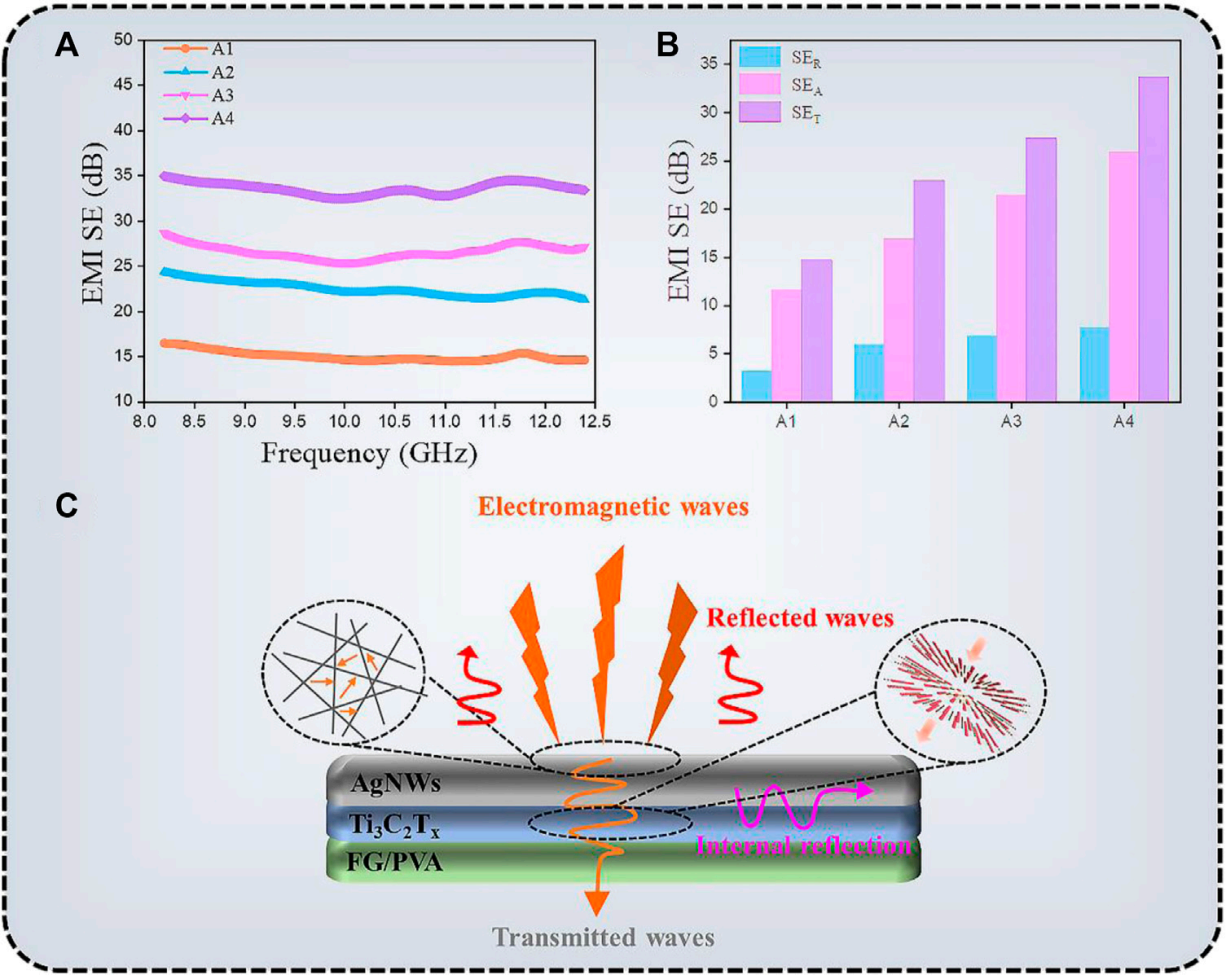
**(A)** EMI shielding performance, **(B)** EMI SE_T_, SE_A_, and SE_R_ values, and **(C)** the mechanism of EMI shielding of AgNWs/Ti_3_C_2_T_x_-FG/PVA films.

Because of the high electrical conductivity of AgNWs/Ti_3_C_2_T_x_-FG/PVA films, it can be used as efficiency transparent EMI shielding materials (Iqbal et al., 2020). The EMI shielding performance of different samples (A1, T_A1_ = ca. 90.2%, R_A1_ = 61.3 Ω/sq.; A2, T_A2_ = ca. 85.1%, R_A2_ = 25.2 Ω/sq.; A3, T_A3_ = ca. 80.2%, R_A3_ = 12.7 Ω/sq.; A4, T_A4_ = ca. 75.1%, R_A4_ = 6.8 Ω/sq.) in X-band (8.2–12.4 GHz) were shown in Figure 8. The measured scattering parameters (S_11_ and S_21_) were used to calculate the power coefficients and the EMI SE using the following equations (Zhou et al., 2020):

$$R={\left|S_{11}\right|}^{2},T={\left|S_{21}\right|}^{2}$$ (3)

1=A+R+T (4)

$$SE_{T}=-10\log\left|T\right|$$ (5)

$$SE_{R}=-10\log\left|1-R\right|$$ (6)

$$SE_{A}=-10\log\left|\frac{T}{1-R}\right|$$ (7)

where *A*, *R*, and *T* are the absorption, reflection, and transmission coefficients, respectively, and *SE*_*T*_, *SE*_*R*_, and *SE*_*A*_ are the total microwave shielding, reflection, and absorption effectiveness, respectively. The average EMI SE values of A1, A2, A3, A4 samples were 15.0, 22.4, 26.6, and 33.6 dB, respectively (Figure 8A). It can be seen that the shielding efficiency of the TCFs increased with the increase of its electrical conductivity. When the transmittance of the TCFs was less than 80%, the shielding performance of the films could meet the requirements of commercial applications (EMI SE value >20 dB). Figure 8B shows the average EMI SE_T_, SE_R_, and SE_A_ value, absorption is the primary mechanism of shielding for TCFs. The scheme of EMI shielding mechanism was shown in Figure 8C.

Because of the massive impedance mismatch at the TCF-air interface, most incident EMWs were reflected when they struck the surface of the AgNWs/Ti_3_C_2_T_x_-FG/PVA film (Zhao et al., 2022). The EMWs interact with the high conductive 3D network, which contribute to ohmic losses, resulting in a drop in energy of the waves. Meanwhile, the good conductive network facilitates the transfer of electrons, making it hard for EMWs to escape from the film until they are dissipated or absorbed in the form of energy. Besides, the large aspect ratio of AgNWs and the sheet structure of Ti_3_C_2_T_x_ make the EMWs internal reflection and scattering multiple times in the conductive network, which further promotes the high absorption attenuation of EMWs.

As is known to all, the working mechanism of the OLED device is to apply positive and negative voltages to the anode and cathode of the device, under the action of the hole and electron in an electric field to the corresponding migration in the organic functional layer, and the light-emitting layer in the composite form excitons, exciton radiative transition that produces light phenomenon. For flexible green light OLED devices based on TCFs, the characteristics of the anode directly affect the performance of the device, including: 1) Good conductivity of the electrode can effectively reduce the device driving voltage; 2) The lower roughness of the electrode can avoid possible short circuits in devices; 3) The higher work function (WF) of the electrode can match the highest occupied molecular orbital (HOMO) energy levels of the hole injection material, which can reduce the potential barrier between the anode and the organic layer, improve the hole injection efficiency, and obtain high-performance devices.

**FIGURE 9:**
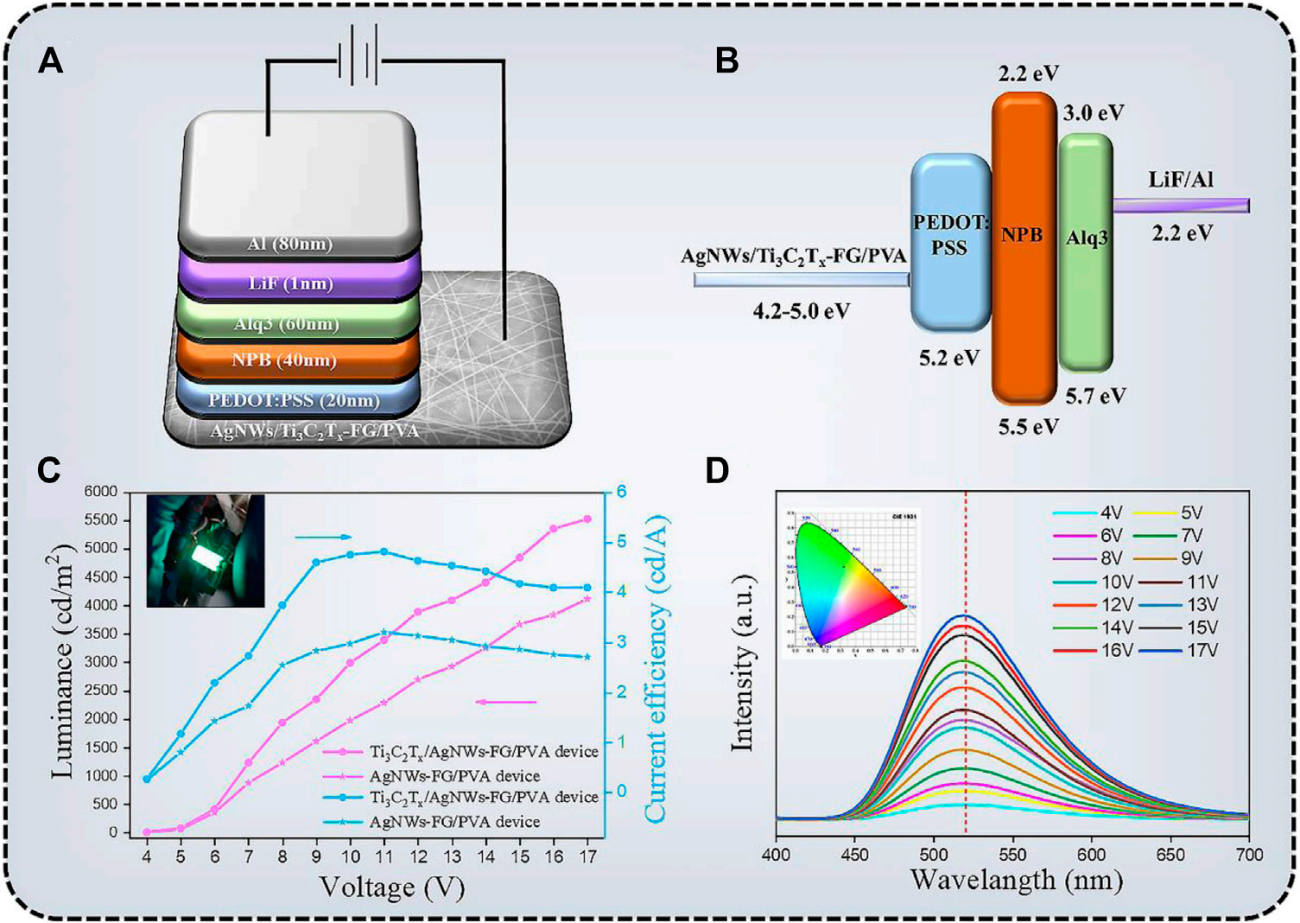
**(A)** The structure of green OLEDs on the AgNWs/Ti_3_C_2_T_x_-FG/PVA anode. **(B)** The energy diagram of the OLED device. **(C)** Voltage-Luminance curve and Voltage-Current efficiency curve for the OLED device (inset: the image of OLED device); **(D)** electroluminescence (EL) spectra of the OLEDs (inset: CIE color coordinates).

The AgNWs/Ti_3_C_2_T_x_-PET FG/PVA TCFs can be used as the anode of flexible OLED due to its flat surface, high optical transmittance, excellent electrical conductivity, and proper WF (Kim et al., 2015). The schematic structure and energy diagram of the device are depicted in Figures 9A,B. PEDOT:PSS (WF = 5.2 eV; *σ* = 1000 s/m) was used as a hole inject layer on AgNWs/Ti_3_C_2_T_x_-PET FG/PVA film, which can not only modify the WF, but also further reduce the roughness of the TCFs. Subsequently, NPB (WF = 5.4 eV), Alq3 (WF = 5.7 eV), and LiF/Al were, respectively deposited on the anode by vacuum evaporation as the hole transport layer, luminescent layer, electron transport layer, and cathode. The relationship between the luminance and current efficiency as a function of voltage for the device are shown in Figure 9C. The maximum luminance of the AgNWs-FG/PVA device was 4,117.3 cd/m^2^ at 17 V, and the maximum current efficiency was 3.21 cd/A at 11 V. In contrast, the AgNWs/Ti_3_C_2_T_x_-FG/PVA device exhibited better performance, which the maximum luminance was 5,525.7 cd/m^2^ at 17 V and the maximum current efficiency was 4.81 cd/A at 10 V. Besides, the external quantum efficiency of the AgNWs/Ti_3_C_2_T_x_-FG/PVA device was shown in Supplementary Figure S6; its maximum external quantum efficiency was 2.55% at 13 V, indicating the good properties of the device. The improved performance of the device was due to the addition of high WF Ti_3_C_2_T_x_ flakes (5.0 eV), which could solve the problem of difficulty in injecting holes from the anode into the organic layer caused by low WF of the AgNWs (4.2 eV). Figure 9D shows the electroluminescence (EL) spectra of the AgNWs/Ti_3_C_2_T_x_-FG/PVA device, the electroluminescence peak of the device was at 520 nm, and with the increase of the driving voltage, the peak of the device does not shift, indicating that the device has very excellent chromatographic purity. The spectrometer PR655 was used to measure the color coordinates of green OLEDs. As shown in the inset of Figure 9D, the color coordinates of the device are shown in CIE 1931 color space (0.30, 0.53). Moreover, the high-performance OLED devices using the degradable AgNWs/Ti_3_C_2_T_x_-FG/PVA film can be degraded in a simple way (as shown in Figure 7) to solve the problem of environmental pollution caused by random disposal of “electronic waste”. This method of preparing degradable OLED devices based on degradable films provides a new idea for the future preparation of environmentally friendly flexible electronic devices.

## 4 Conclusion

In summary, we demonstrated a method for preparing flexible TCFs based on naturally degradable material-FG, in which AgNWs and Ti_3_C_2_T_x_ flakes with excellent electrical conductivity were used as conductive fillers. The film has low roughness, good photoelectric properties, strong interfacial adhesion, and degradability. The good properties of the film are the guarantee of its wide application in various fields. The film showed a certain shielding performance in the field of EMI shielding, in addition, the film can be used as an anode to prepare flexible high-performance OLED device. All the evidence confirmed the potential application value of this new type of TCFs in various fields, and its degradability would alleviate the environmental pollution problems caused by the iteration of electronic products.We believe that these TCFs will shine in the smart wearable field in the future.

## Data Availability

The original contributions presented in the study are included in the article/Supplementary Material, further inquiries can be directed to the corresponding authors.
