# Supplementary material for: Environment-friendly AgNWs/Ti3C2Tx transparent conductive film based on natural fish gelatin for degradable electronics
Source: Front Chem. 2022 Aug 5;10:973115. doi: 10.3389/fchem.2022.973115 (PMC9388723; doi:10.3389/fchem.2022.973115)
Supplement: Supplementary file 1 [file DataSheet1.docx]

Environment-friendly AgNWs/Ti_3_C_2_T_x_ transparent conductive film based on natural fish gelatin for degradable electronics

Yuzhou Wang^1,2,3^#, Tao Wang^3,4^#, Yan Liu^2^, Hong-Zhang Geng^3*^, Lianzhong Zhang^1*^

^1^Henan Engineering Technology Research Center of Ultrasonic Molecular Imaging and Nanotechnology, Henan Provincial People’s Hospital, People’s Hospital of Zhengzhou University, Zhengzhou, China

^2^ College of Materials Engineering, Henan University of Engineering, Zhengzhou, China

^3^ Tianjin Key Laboratory of Advanced Fibers and Energy Storage, School of Material Science and Engineering, Tiangong University, Tianjin, China

^4^ Sinopec Petroleum Engineering Zhongyuan Corporation, Zhengzhou, China

# These authors contribute equally.

Supplementary Material


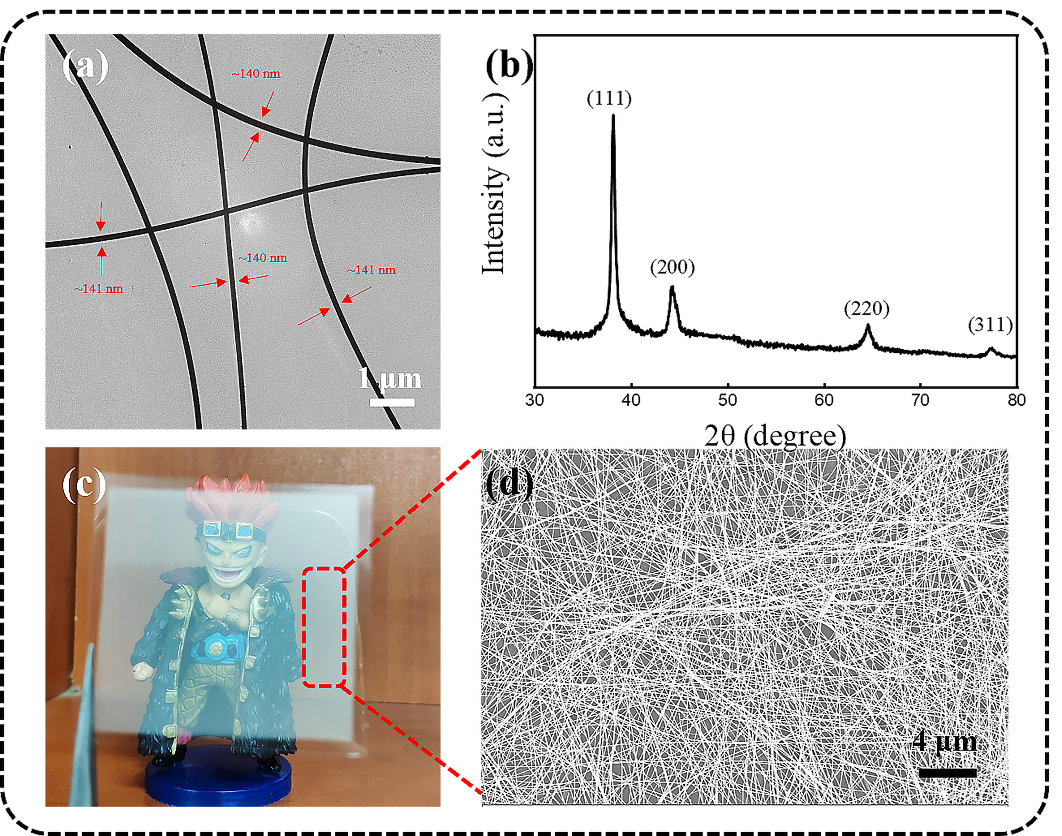


**Supplementary Fig. 1.** TEM images (a) and XRD spectra (b) of the fabricated AgNWs by simple polyol process method. (c) Photograph of the AgNWs film deposited on a PET substrate, SEM images of AgNWs- PET film.

The AgNWs were fabricated by the simple polyol process method. As shown in Fig. S1a, the average diameter and lengths of pure AgNWs were ~140 nm and 40-50 μm. The high aspect ratio (360) of AgNWs was beneficial to form efficient conductive networks. As for the XRD spectrum of AgNWs (Fig. S1b), the four strongest peaks were observed at 38.2, 44.4, 64.6, and 77.5°, which were attributed to the diffraction of the (111), (200), (220), and (311) crystalline planes of the face-centered structure of silver, respectively; and no silver oxide peaks appeared. This indicated the high quality of the fabricated nanowires. For pure AgNWs TCF, the AgNWs film also showed good transmittance (Fig. S1c), and the nanowires overlap each other and evenly distributed on the PET substrate (Fig. S1d).


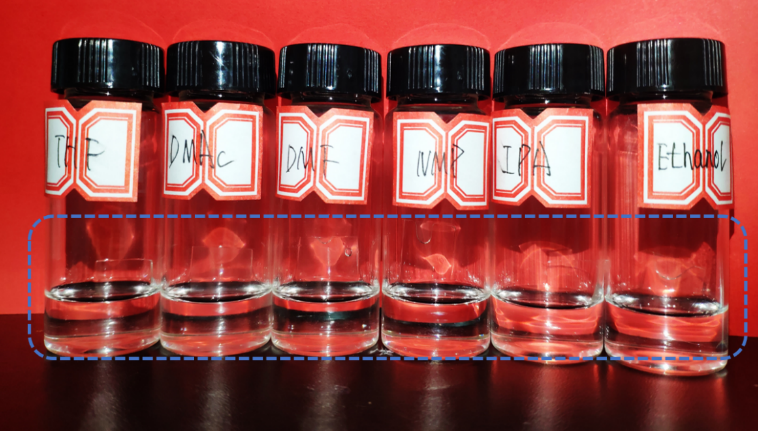


**Supplementary Fig. 2.** Photographs of the FG/PVA films after being immersed in different organic solvents for 24 h: THF, DMAC, DMF, NMP, and IPA.


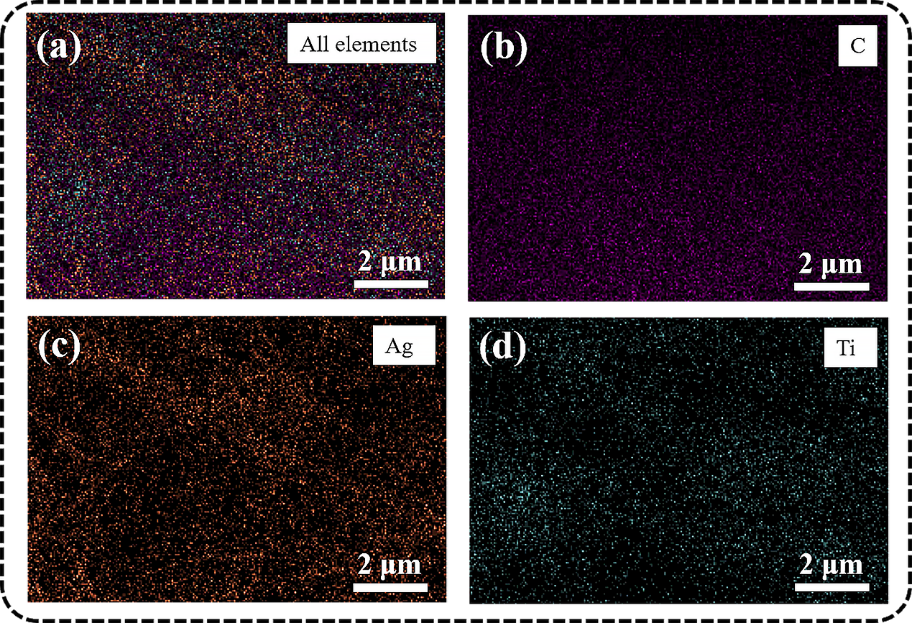


**Supplementary Fig. 3.** Elemental mapping of AgNWs/Ti_3_C_2_T_x_ -PET film.


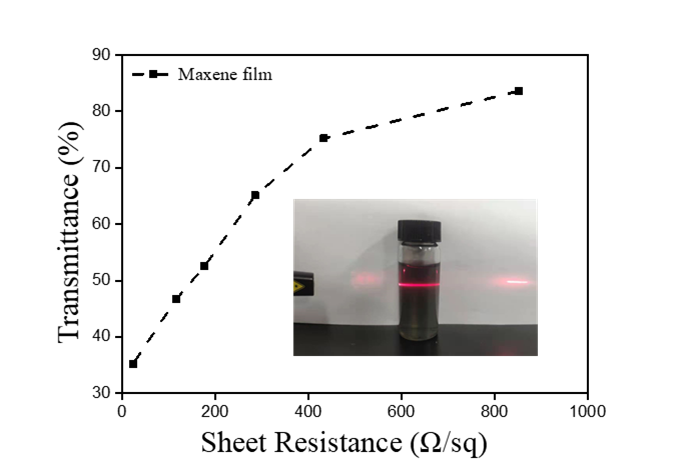


**Supplementary Fig. 4.** The characteristic resistance of the Ti_3_C_2_T_x_-PET film

**Supplementary** **Fig. 5.** The sheet resistance change of AgNWs-PET, AgNWs/Ti_3_C_2_T_x_-PET, AgNWs/Ti_3_C_2_T_x_-FG/PVA films as a function of bending cycle number.

**Supplementary** **Fig.6.** The external quantum efficiency of the AgNWs/Ti_3_C_2_T_x_-FG/PVA device
